# The Art of Revising

**DOI:** 10.5334/pme.1073

**Published:** 2023-06-20

**Authors:** Chris Watling

**Affiliations:** 1 Western University, Canada

## Abstract

In the writer’s craft section we offer simple tips to improve your writing in one of three areas: Energy, Clarity and Persuasiveness. Each entry focuses on a key writing feature or strategy, illustrates how it commonly goes wrong, teaches the grammatical underpinnings necessary to understand it and offers suggestions to wield it effectively. We encourage readers to share comments on or suggestions for this section on Twitter, using the hashtag: #how’syourwriting?

*We read your paper with great interest. Unfortunately, we are unable to accept it in its current form. However, we would be pleased to consider a revision that addresses the issues raised by the reviewers*.

Sound familiar? Any time your paper elicits this response from a journal – a category I like to call “not rejected” – it’s a cause for celebration. But emails like this often land with more of a thud than celebratory fanfare. While you may be a step closer to publication, you’re going to have to work for it.

Revising a paper into which you’ve already poured your heart and soul presents a tough writing challenge. The difficulties are technical, intellectual, and emotional. And the stakes are high. In this Writer’s Craft, I offer some strategies to help you make it over this final hurdle to publication of your work. To illustrate these strategies, I offer examples of responses to reviewers and of revisions themselves. Because revision is not a public process, I have only my own examples to draw on; in sharing them, I hope to pull back the curtain on the private work of revising a manuscript.

## Let the dust settle

Papers almost never get accepted without authors needing to revise them. Still, facing reviewer comments can be disappointing, anxiety-provoking, or infuriating. Sometimes they make us wonder whether we had any business submitting our work in the first place.

These reactions are normal, but they don’t exactly nurture the optimal mindset from which to approach a revision. Most of us do better if we let the emotional dust settle before approaching the task of revising. So, take a few days, and then return with a clear head and a mended heart. Remember that revising is just part of the job of a researcher.

The work of revising is not emotionally neutral, however, and advice to approach the task “objectively” oversimplifies the challenge. Instead of aiming for unrealistic detachment, I suggest remaining attentive to your emotional reactions throughout the process. The goal is to ensure that your emotional connection to your work doesn’t blind you to the real opportunities this process offers to strengthen your story and heighten its impact.

Try to remember that an invitation to revise, especially in this era of low acceptance rates, is a gift. As one journal editor noted, “By virtue of inviting you to revise and resubmit, we are investing in you, we want to see you be successful. The last thing we want to do is discourage you from writing [1].”

## Lean on your team

In my experience, it is tough to write revisions as a team. Someone – typically the first author – needs to take the lead. But that doesn’t mean the rest of the team are passive; they can be critical facilitators of a successful revision. Lean on your team to help you manage your emotions. Vent a bit to them so that anger, bitterness, or defensiveness don’t seep into your response to the journal. Rely on the calm distance that a less-emotionally-invested middle author can sometimes offer. Engage your team to help you to interpret reviews that are unclear and to suggest options for addressing them. Seek your team’s perspective on whether a response is too cheeky, a revision too superficial, a statement of limitations too defensive.

## Engage meaningfully

When a journal’s editor offers you an opportunity to revise, they expect you to engage meaningfully with reviewers’ comments. Don’t disappoint them with a half-hearted effort. You needn’t agree with every reviewer comment, but you do need to treat each with respect.

Sometimes engagement is easy. When reviewers plead for greater clarity, you should always try to deliver. It is never sufficient to say “We believe we have already addressed this issue clearly in paragraph 1 of our Methods section, and so we have made no changes.” Instead, reframe the comment as an opportunity to avert a potential misunderstanding:

*We thank the reviewer for alerting us to this lack of clarity in our Methods section. We have rewritten the paragraph with more explicit attention to the sequencing of the steps in our work to make it clearer how the research unfolded*.

Sometimes engagement is more challenging, as it requires you to re-examine the very foundations of your work. When a reviewer points you toward a paper, a book, or a whole body of literature they see as relevant, you’ll need to read it critically, consider its application to your work, and *show* the fruits of that labour in your revision. Recently, for example, a reviewer suggested that our authorship team incorporate theories of human agency into our introduction. Of course, such theories are abundant in the literature, and this request initially felt overwhelming. Ultimately, our solution was to engage meaningfully with some key literature, while avoiding a rabbit hole, as we described in our response to reviews:

*We have made an explicit link to Bandura’s social cognitive theory in our introduction of the notion of agency. We hope this approach better grounds the work in broader theories of agency without introducing too many theoretical perspectives that would risk losing focus*.

When a reviewer offers up a highly relevant paper, don’t waste the opportunity to strengthen your manuscript by merely adding it to your reference list. Use it to shore up your argument or to connect ideas. For example, in the Introduction of a paper exploring how research authors navigate the peer review process, we outlined several known influences on how individuals interact with feedback in other contexts, but a smart reviewer directed us to a key paper we had missed. We reviewed the paper and agreed that it was not only relevant, but also better aligned with the peer review context than other literature that we had cited. In our revision, we featured it early in the paragraph that reviewed this literature, and we explicitly showed how the paper (described in the revised manuscript below as a “recent realist review”) was relevant to the peer review context we were studying:

*Numerous influences on how individuals interact with feedback in other contexts have been described. A recent realist review of feedback interventions for written tasks in higher education offers useful insights, given the similarity between a student’s submission of an assignment for feedback and grading and a researcher’s submission of a manuscript for review. This review identified two key influences on how feedback works…* [2]

Above all, remember the obvious – you need to actually revise the manuscript. Sometimes, the energy authors put into their “response to reviews” document is not reflected in meaningful changes to the paper. If your responses are elaborate but the changes you make are minor, your engagement in the process may be perceived by the editor as superficial. Remember that acceptance of your revised manuscript is not guaranteed, and try to avoid offering the editor an easy justification to “reject after revision.”

## Elaborate and acknowledge

Critiques of your study’s design can be challenging. If a reviewer asks questions about your methodology, these questions are likely to occur to future readers. Your revision, therefore, should invite readers more fully into your research process. In a paper exploring coaching within and outside of medical education, my co-author and I received requests from two reviewers for additional insights into *how* we enacted reflexivity. We recognized that the paragraph we had devoted to reflexivity in our Methods was limited to describing ourselves and our orientation to the research question, and we embraced the opportunity to elaborate. But we also worried about how we could do justice to the reviewers’ comments without exploding our word count. We reflected this concern in our response to reviews:

*It is challenging within the word count to elaborate all the ways that reflexivity played out, but we thank the reviewer for pushing us to make this more explicit. We have added a few sentences to the second-last paragraph of the Methods section to give the reader a stronger flavor of the way our reflexivity shaped our analysis*.

And in the revised manuscript, the “few sentences” we referred to in our response were these:

*We considered whether our data reinforced or upended our assumptions about coaching, and engaged in regular discussions with each other about our evolving perspectives. For example, we were surprised by sports coaches’ stronger emphasis on the development of their athletes as people than on their athletic success. Reflecting on how this data challenged our assumptions about sports coaching led us to wonder how such a holistic, learner-centred approach would fit in a patient-centred clinical environment* [3].

If a reviewer takes issue with design decisions that you cannot change, engage by looking critically at how you describe your study’s limitations. Have you both acknowledged the methodological concerns that rankled the reviewers and reflected on their impact? In this same coaching study, one reviewer raised concerns about how our sampling strategy impacted our results and analysis. They highlighted a sense of idealism pervading many of the quotes we shared, and asked whether our self-selected sample had produced an overly positive and rather untroubled conceptualization of coaching. Their concerns were not unreasonable; we too had been struck by the learner-centred and idealistic way that many of our participants had spoken about coaching. While we couldn’t revisit our sampling strategy, we did engage more robustly in considering its consequences in our revised Limitations paragraph, to which we added these sentences:

*That our participants consistently reflected a learner-centred approach to coaching is almost certainly influenced by our sampling strategy; coaches of professional sports teams might well have instead invoked winning and profit as motivators…Finally, we recognize that our participants tended to discuss coaching in idealistic terms; individuals who would choose to participate in a study about coaching perhaps harbor a distinctly positive orientation toward coaching that is not universally held* [3].

## Lighten your grip

You need to pick your battles. I received a review recently in which the reviewer commented that they found my use of the verb “eschew” to be pretentious. Meaning-wise, the verb was appropriate for the sentence, and my first instinct was to try to explain to the reviewer why the verb was exactly right. But once I got over my writerly hubris, I realized that any perception of pretentiousness in the writing might threaten its accessibility. With many plain language alternatives at my disposal, like “avoid” or “cast aside” or “dismiss”, I decided not to fight a battle that wasn’t worth winning. And the sentence was more inviting as a result.

Reviews are a powerful preview of your intended audience’s likely reaction to your work. If most or all reviewers find something confusing, then it needs to be rewritten. If reviewers find a table or figure doesn’t add clarity to your argument, take it out, even if you worked hard to create it. If your title gives reviewers the wrong impression about the paper’s key message, change it, even if you find it compelling. To guard against what Taylor calls “inexplicable stubbornness [4],” lighten your grip on specific words or phrases and maintain emotional control throughout the process of revising.

Sometimes a turn of phrase to which you’ve become attached actually threatens your manuscript’s message. Reviewer reactions are a useful signal of this risk, flagging places where you have inadvertently opened a door that you had intended to keep closed. Perhaps you’ve created expectations you don’t intend to deliver on or raised questions you don’t intend to answer. Quite often, the root of the problem is a cavalier choice of words, or a phrase chosen more for its style than its substance. Reconsidering your word choice can be an easy fix. For example, the Results section of our coaching paper originally opened with this sentence:

*We identified a shared philosophy of coaching, comprising a number of core elements that our participants endorsed regardless of the coaching context*.

One reviewer thought this sentence suggested that we had asked participants to confirm a pre-determined framework for coaching, which they (correctly) felt was incompatible with our inductive research methodology. We speculated that the root of the problem was our use of the verb “endorsed”, with its unintended implication of a verification process. So, we changed it in the revised manuscript:

*We identified a shared philosophy of coaching, comprising a number of core elements that seemed to anchor participants’ approaches to coaching, regardless of context* [3].

This example reminds us of the power of even individual words to lead readers astray. Some words and phrases immediately create expectations for readers, and we need to be meticulous about ensuring that we’re creating the ones we intend. For example, in a paper exploring learner agency, our Discussion originally included this line:

*In order to exercise agency in ways that would lead to support rather than sanction, they [learners] had to first earn and establish social capital*.

Our sloppy use of the term “social capital” created unintended expectations in a reviewer, who commented “having social capital and being a strong learner get conflated and I am not sure that they mean the same thing.” While the reviewer asked us to better differentiate these ideas, we recognized that we should never have opened this door in the first place, and we revised this way:

*In order to exercise agency in ways that would lead to support rather than sanction, they had to first earn the trust and confidence of their teachers and supervisors* [5].

The lesson here is to remain rather loosely attached to your word choices, maintaining an openness to changing your phrasing when reviewer comments signal that its hoped-for effect has not been realized.

## Resist when necessary

You needn’t bend to everything that reviewers suggest. Judicious resistance to some reviewer comments is not only acceptable, but also expected of a researcher. Stand your ground in places where it really matters to the integrity of the work, but avoid the trap of defensively rejecting most of the reviewer suggestions.

Sometimes, reviewers ask for things you may not be willing or able to deliver. For example, reviewers may want detailed demographic information about participants in a qualitative study to help them to better contextualize your results. Your ethical obligation to protect your participants by avoiding potentially identifying information, however, trumps reader curiosity. But your response still needs to indicate engagement with the reviewer’s reasons for wanting the information. When I receive this kind of comment, I consider whether I can offer something that meets them part way without compromising ethical obligations. For example, here’s a response to a recent reviewer request for detailed demographic information, including career stage, about our participants:

*We reflected on providing more identifying information but have decided not to do so for two reasons: 1) we committed to protecting the confidentiality of our participants, and so we are reluctant to provide additional demographic information for each quote; 2) career stage is a somewhat slippery concept when it comes to publishing research, as some individuals were well into a clinical career but relatively inexperienced as published researchers, for example. We have, however, added a nod to career stage for three quotes for which we think that knowing that the quote came from a senior researcher is particularly salient to its interpretation*.

As this example shows, effective resistance requires that you engage with the reviewer’s idea and offer a sound rationale for your choice not to adopt it. It isn’t sufficient to simply say “We disagree and have not altered the text.” Disagreement needs to be reasoned and diplomatic. Engaging with the reviewer’s idea helps in this regard, especially when you can incorporate elements of that idea in your revision even if you don’t adopt it wholesale. The wording of your response can also help; focusing on your rationale for resisting a change is typically more diplomatic than critiquing the reviewer. For example, a reviewer of a recent paper asked us to engage more deeply with the literature on autonomy, but we did not feel that doing so would strengthen the paper. In our response to reviews, we offered a modest compromise while describing our thought process in not going further:

*We have added a brief elaboration of the notion of autonomy, including a citation of a recent paper on the issue. We worry that a more detailed exploration of autonomy would risk distracting readers from the main thrust of the Introduction, which is to examine what we already understand about agency*.

The use of “We worry…” offers more diplomacy than “The reviewer’s comment risks creating a distraction.”

Especially challenging is the situation of conflicting or contradictory reviews, in which case resistance to at least some of the reviews is unavoidable. When reviews conflict, try to act on the reviewer suggestions that strengthen your paper’s essential messages, and explain clearly in your response why you have chosen to follow one reviewer’s suggestions over another’s. And if you can’t resolve the quandary this way, you can always ask the editor. No revision was ever rejected because the author reached out to the editor for clarity.

Finally, it’s useful to remember that the journal and its editor will have some non-negotiables of their own; on these issues, resistance will be futile and will likely lead to rejection of the paper. LaPlaca helpfully reminds us that not all reviewer comments are created equal; some revisions are “must-dos”, and publication will likely depend on a convincing response from you [6]. Your challenge is to identify which comments fall into this category and to ensure that they receive careful attention. Concerns expressed by more than one reviewer, issues reinforced by the editor’s own comments in their email to you, and reservations expressed about whether your work adds anything new to the literature are examples of signals you shouldn’t ignore. This issue of novelty or originality can be especially critical; its appearance in reviews suggests a worrying ambivalence about your paper that you can’t afford to disregard.

## Focus on flow

Revisions risk disrupting the flow and logic of your writing. You’re never really revising a sentence or a paragraph or a section; you’re revising the whole paper. It will be a different product when you are finished – almost certainly a better product – and you’ll need to ensure that it continues to hang together as a cohesive research story.

Consider how best to make space for a revision. In our paper on the peer review process, reviewers asked for more attention to practical implications of our work. Our original Discussion was primarily focused on theoretical matters, so we crafted a new paragraph in the revision that we set off with a subheading (“Implications”) to make it unmissable. And to lead off this section, we borrowed the first three sentences of what had been our original Conclusion section, because we thought they offered a nice transition from the theoretical to the practical:

*Peer review occupies a powerful cultural position within academia. Researchers who wish to publish their work **must** engage. Journals and the academics who review for them thus have a responsibility to shape a culture of peer review feedback that is as consistently productive as possible. To this end, we offer some practical suggestions* [2].

Of course, we then had to re-tool our Conclusion so it wasn’t redundant. We did so in a way that allowed us to pivot back to our overarching aim of using the peer review setting to shine a light on feedback as a tricky exchange, regardless of context:

*Peer review offers challenges to the exchange of meaningful feedback. But no feedback situation is perfect…*. [2]

Not all revisions require an entirely new paragraph or section to be written; often you can succeed by adding material to an existing paragraph. Remember that new material alters the focus of a paragraph, even if it is highly germane to your argument. Pay careful attention, therefore, to paragraphing principles [7]. Ask whether your topic sentence remains appropriate to your revised paragraph. Ask whether the paragraph maintains unity and coherence. And ask whether transitions from the preceding paragraph and to the following paragraph require revisiting or strengthening.

## Conclusion

Revising a manuscript is arduous work. But peer review provides a window on how readers will react to what you have written. This critical preview is a valuable opportunity to clarify or correct, to mitigate misinterpretation, and to elaborate connections to existing work. Relying on a few key strategies can help you to make the most of this opportunity: use your team, engage meaningfully, elaborate and explain your thinking, remain open to change, and resist judiciously.

An offer to revise represents a chance to reach higher than we initially aimed. That is a gift indeed.
